# Association Between Maximum Lifetime Body Mass Index and Nailfold Capillary Changes in Patients With Type 2 Diabetes Mellitus

**DOI:** 10.7759/cureus.75411

**Published:** 2024-12-09

**Authors:** Maiko Shikama, Sayaka Suga, Tetsuya Tajima, Junji Kozawa, Norikazu Maeda, Michio Otsuki, Taka-Aki Matsuoka, Iichiro Shimomura, Yuko Ohno

**Affiliations:** 1 Department of Fundamental Nursing, Shiga University of Medical Science, Shiga, JPN; 2 Department of Nursing, Graduate School of Health Sciences, Kobe University, Kobe, JPN; 3 Department of Mathematical Health Science, Graduate School of Medicine, Osaka University, Osaka, JPN; 4 Department of Metabolic Medicine, Graduate School of Medicine, Osaka University, Osaka, JPN; 5 Department of Endocrinology, Metabolism, and Diabetes, Faculty of Medicine, Kindai University, Osaka, JPN; 6 Department of Endocrinology, Tokyo Women’s Medical University, Tokyo, JPN; 7 First Department of Medicine, Wakayama Medical University, Wakayama, JPN

**Keywords:** microvascular dysfunction, nail fold capillaroscopy, nail fold capillary, obesity history, past obesity

## Abstract

Objective: Microvascular changes, such as crossing nailfold capillaries, could be crucial for linking maximum lifetime body mass index (BMI) and microvascular complications in patients with type 2 diabetes mellitus (T2DM). However, the relationship between maximum lifetime BMI and microvascular changes remains elusive. This study aimed to elucidate the relationship between maximum lifetime BMI and the percentage of crossing nailfold capillaries among patients with T2DM.

Methodology: As an extension of a cross-sectional study at Osaka University Hospital, this study was conducted among 63 patients with T2DM aged 40-75 years. Maximum lifetime BMI data were extracted from medical records, and nailfold capillaroscopy was applied to assess capillary morphology, following the simple capillaroscopic definitions established by the European Alliance of Associations for Rheumatology Study Group. The association between maximum lifetime BMI and percentage of crossing fingernailfold capillaries was evaluated using multiple linear regression, adjusting for potential confounders such as age, sex, obesity status at the time of the survey, and other diabetes-related factors.

Results: After adjusting for confounding factors, maximum lifetime BMI was significantly correlated with higher crossing capillary percentage (standardized regression coefficients: 0.47; *P *= 0.026). BMI at the time of the survey showed no significant association with the percentage of crossing capillaries (standardized regression coefficients: -0.21; *P* = 0.381).

Conclusions: Maximum lifetime BMI was associated with a higher percentage of crossing capillaries in patients with T2DM, rather than obesity status at the time of the survey. These results emphasize the importance of lifelong weight management in the prevention of T2DM and its complications and highlight the necessity of considering maximum lifetime BMI alongside current BMI in the management of patients with T2DM.

## Introduction

With the global increase in the prevalence of obesity, the number of patients with type 2 diabetes mellitus (T2DM) and its complications is also rising [1]. Maximum lifetime body mass index (BMI) is considered a validated summary measure of an individual’s obesity history [2], and recent studies have shown that current BMI and maximum lifetime BMI increases the risk of developing microvascular complications in patients with T2DM [3-6]. Obesity causes changes in the microvasculature even before the onset of T2DM; these changes are believed to induce insulin resistance and hypertension, contributing to the development of T2DM and its microvascular complications [7,8]. Therefore, microvascular changes may be a crucial link between maximum lifetime BMI and microvascular complications in patients with T2DM. However, the relationship between maximum lifetime BMI and microvascular changes has not yet been thoroughly investigated [9].

Nailfold capillaries are critical sites where changes in the microvasculature can be assessed non-invasively and visually and are currently used in the early diagnosis of conditions such as systemic sclerosis [10]. Among the morphological changes in nailfold capillaries, crossings are indicators associated with T2DM and its complications [10]. Previous studies revealed that patients with T2DM have more crossings in their nailfold capillaries compared to healthy individuals, and individuals with diabetic complications have more crossings in their nailfold capillaries than their counterparts without complications [11-15]. Notably, crossing capillaries in the nail fold are associated with an increased risk of diabetic retinopathy and nephropathy in patients with T2DM [12,13]. The relationship between current obesity status and the percentage of crossing nailfold capillaries in patients with T2DM has been examined previously [16]; however, the relationship between maximum lifetime BMI and the percentage of crossing capillaries has not been examined.

Therefore, the aim of this study was to examine the relationship between maximum lifetime BMI and the percentage of crossing nailfold capillaries in patients with T2DM.

## Materials and methods

Study population

**Figure 1:**
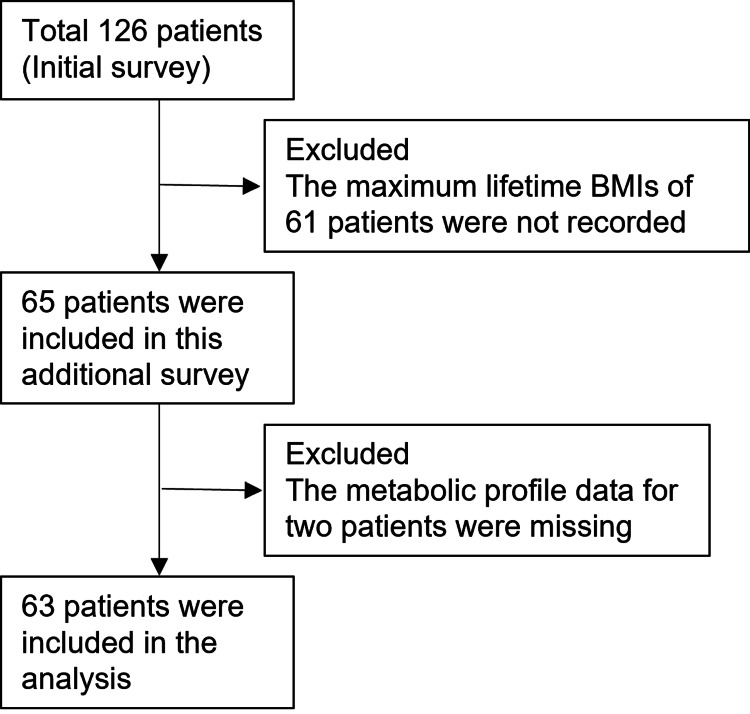
Patient flow diagram. BMI, body mass index

This study was conducted in March 2024 as an additional survey following a cross-sectional study that investigated the association between morphological abnormalities of nailfold capillaries and diabetic complications, as well as the factors influencing morphological abnormalities of nailfold capillaries in patients with T2DM aged 40 to 75 years, who visited the Department of Metabolic Medicine at Osaka University Hospital between May 9, 2019, and October 10, 2019. In this additional survey, maximum lifetime weight data, which were not included in the initial study, were retrospectively collected from medical records to examine their association with nailfold capillary crossing. Therefore, no formal sample size calculation was performed, as the study utilized existing data. Some of the data in this cross-sectional study have been used in other studies for analysis [12,16]. The exclusion criteria included a history of Raynaud’s phenomenon; the presence of collagen tissue disease, glaucoma, uveitis, dementia, cerebrovascular disease, coronary artery disease, dialysis, and blindness; undergoing cancer treatment; current smoking status; removal of cuticles; use of nail polish within 1 month before the test; and pregnancy. Among the 126 participants involved in the initial survey on nailfold capillaries, 65 patients were included in this additional survey owing to missing data on maximum lifetime weight (Figure 1). This study was approved by Osaka University’s Institutional Review Board on April 9, 2019 (approval no. 18546) and was conducted in adherence to the ethical principles outlined in the Declaration of Helsinki and its subsequent revisions. All participants provided written informed consent.

Obesity index

Maximum lifetime weight data were obtained from the patient’s medical records. The medical staff measured the height, weight, and waist circumference (WC) of the patients. BMI was calculated as weight (kg) divided by height squared (m^2^). Maximum lifetime BMI was calculated using values of maximum lifetime weight from the medical records and height measured during the survey. BMI at the time of the survey was calculated from the measured height and weight. WC at the time of the survey was measured using a non-elastic metric tape at the umbilical level at the end of the exhalation phase, with the patient standing and exhaling.

Outcome

Details of the methods for photographing and evaluating nailfold capillaries have been reported elsewhere [12,16]. Imaging of nailfold capillaries was performed using a GOKO Bscan-Z microscope (GOKO Imaging Devices Co., Kanagawa, Japan) at a magnification of ×390 by a trained technician who was unaware of the participant’s medical condition. Three images of the central part of the nail folds of each finger, excluding the thumbs, were taken [17]. The dimensions of the capillaroscopy image were approximately 0.5 × 0.7 mm^2^. Two blinded raters analyzed the capillaries in the distal row of the nail fold. A typical capillary appears as a hairpin or an inverted *U* letter, comprising an arterial segment, a top section, and a venous segment [18]. The nailfold capillary morphology was classified as *crossing* (the limbs crossed once or twice, and the capillary head was convex) or other types according to the simple capillaroscopic definitions of the European Alliance of Associations for Rheumatology Study Group on Microcirculation in Rheumatic Diseases [19,20]. This definition was created to simplify and standardize the morphological findings of nailfold capillaries, and it has excellent reliability [20]. In this study, the inter-rater reliability of nailfold capillary morphological assessment was excellent (Cohen’s κ, 0.84). The percentage of crossing capillaries was determined by dividing the number of crossing capillaries by the overall number of capillaries evaluated.

Demographics and clinical characteristics of patients

Medical staff performed all the measurements. Blood pressure was measured twice on the same arm using a digital blood pressure machine (HEM-8713; Omron Corporation, Kyoto, Japan), and the average of the two measurements was used. Information regarding sex, age, presence or absence of diabetic retinopathy, urine findings, estimated glomerular filtration rate (eGFR), high-density lipoprotein cholesterol (HDL-C) levels, low-density lipoprotein cholesterol (LDL-C) levels, triglyceride (TG) levels, most recent glycated hemoglobin (HbA1c) value, duration of diabetes, insulin treatment, and medication use (antihypertensive and antilipidemic medications) were collected from the patient’s medical records. Dyslipidemia was defined as antilipidemic medication use, HDL-C level <40 mg/dL, LDL-C level ≥140 mg/dL, and/or TG level ≥150 mg/dL [21]. Diabetic nephropathy (overt nephropathy or kidney failure) was defined as follows: urine albumin-to-creatinine ratio ≥300 mg/g creatinine or urinary protein-to-creatinine ratio ≥0.5 g/g creatinine or positive proteinuria by dipstick analysis examination, or eGFR <30 mL/min/1.73 m^2^ [22]. Diabetic retinopathy (simple diabetic retinopathy, pre-proliferative diabetic retinopathy, or proliferative diabetic retinopathy) was defined according to Davis’s classification [23]. Hypertension was defined as antihypertensive drug use, systolic blood pressure (SBP) ≥140 mmHg, and/or diastolic blood pressure (DBP) ≥90 mmHg [24]. Regular exercise was defined as engaging in workouts at least twice a week, with each session lasting a minimum of 30 minutes over no less than six months [25]. Metabolic syndrome was defined based on the Japanese Society of Internal Medicine criteria [26] and was diagnosed when a patient had an increased WC (≥85 and ≥90 cm for male and female, respectively) and at least two of the following three additional components: (1) impaired glucose metabolism, defined as fasting blood glucose levels ≥110 mg/dL and/or HbA1c value ≥6.0%, or medication use for diabetes; (2) elevated blood pressure, defined as SBP ≥130 mmHg and/or DBP ≥85 mmHg, or antihypertensive drug treatment; and (3) elevated TG levels (≥150 mg/dL or drug treatment for elevated TG levels) and/or decreased HDL-C levels (<40 mg/dL or treatment for decreased HDL-C levels).

Statistical analyses

Patient characteristics according to maximum lifetime BMI tertiles were compared using the Cochrane-Armitage and Jonckheere-Terpstra tests for trend for proportions and continuous variables, respectively. Crossing capillary percentages according to maximum lifetime BMI are graphically presented as a scatter plot. General linear models were used to evaluate the association between maximum lifetime BMI and percentage of crossing finger nailfold capillaries, adjusting for potential confounders: age, sex, regular exercise (yes/no), duration of diabetes, HbA1c levels, DBP, renin-angiotensin system inhibitor use (yes/no), lipid-modifying medication use (yes/no), and BMI or WC at the time of the survey. The relative contributions of these variables to the percentage of crossing capillaries were compared by estimating the standardized regression coefficient. We ensured that these variables were appropriately scaled and met the assumptions required for linear modeling. For all statistical analyses, a two-sided *P*-value <0.05 was considered significant. All data analyses were performed using IBM SPSS Statistics, Version 24.0 (IBM Corp., Armonk, NY).

## Results

Among the 65 participants, two were excluded due to incomplete metabolic profile data, yielding a final analytical sample of 63 patients (Figure 1). The participants had a mean age of 62.9 years with a standard deviation (SD) of 8.9, and 47.6% of them were female. On average, each patient had 91.1 nailfold capillaries (SD = 26.8), with a mean percentage of crossing capillaries of 62.3% (SD = 11.4).

**Figure 2:**
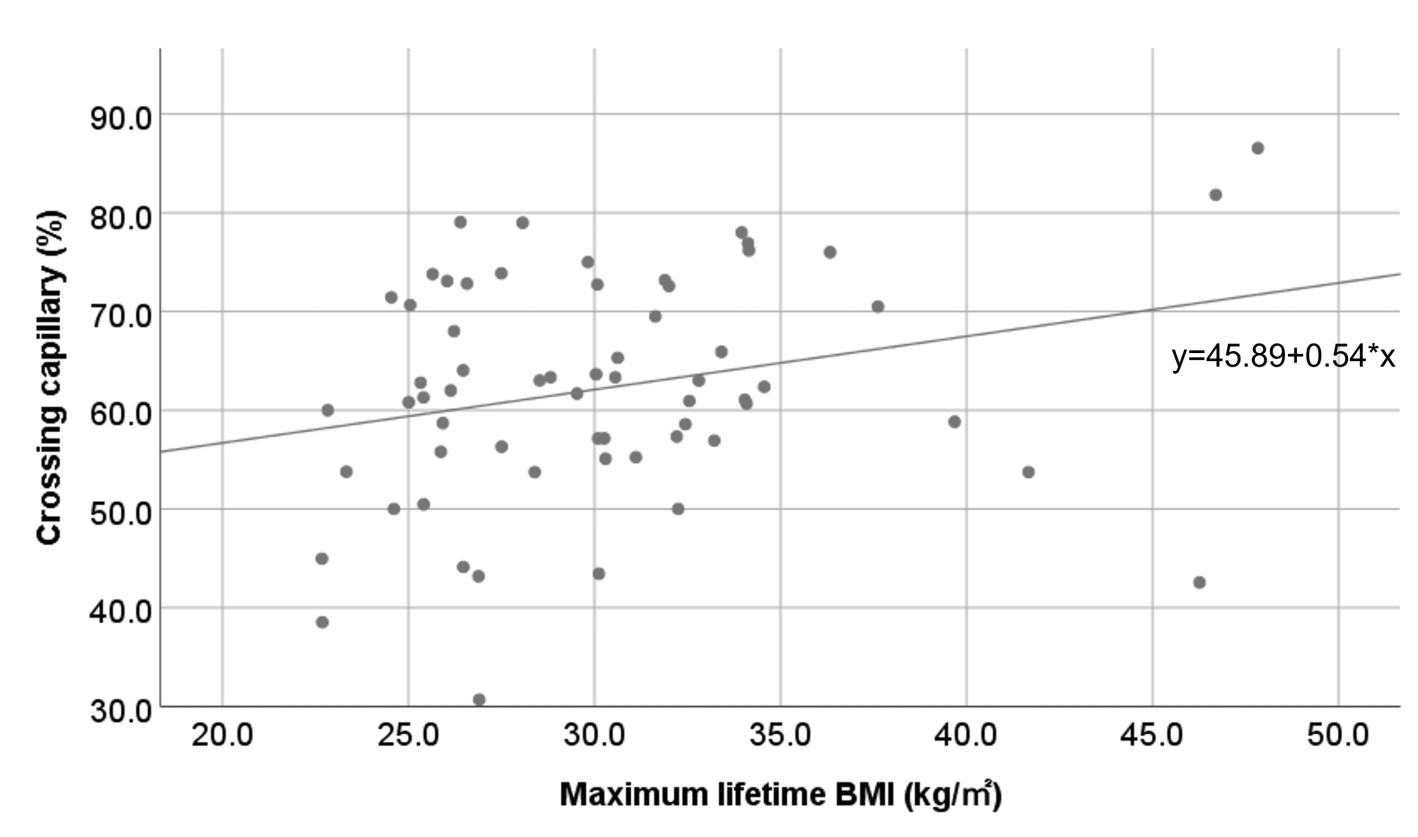
Scatter plot of maximum lifetime BMI and percentage of crossing capillaries. The best-fit line represents a linear regression. BMI, body mass index

Table 1 shows the participants’ demographic and clinical characteristics according to the corresponding maximum lifetime BMI tertiles. Age displayed a significant reduction with increasing maximum lifetime BMI values. At the time of the survey, DBP, BMI, and WC indicated a significant increase with increasing maximum lifetime BMI values (P for trend < 0.05). Figure 2 is a scatter plot showing a positive relationship between maximum lifetime BMI and the percentage of crossing capillaries. This plot highlights the linear increase in capillary crossing with increasing maximum lifetime BMI.

**Table 1:** Clinical characteristics according to maximum lifetime BMI. Continuous data were analyzed using the Jonckheere-Terpstra test for trend and are presented as mean ± standard deviation. Categorical data were analyzed using the Cochrane-Armitage test for trend and are presented as %. ^a^Crossing capillary (%) was calculated by dividing the number of assessed crossing capillaries by the number of assessed nailfold capillaries. ^b^Diabetic nephropathy was assessed using the Classification of Diabetic Nephropathy. ^c^Diabetic retinopathy was defined according to the Davis classification. ^d^Hypertension was defined as systolic blood pressure ≥140 mmHg, diastolic blood pressure ≥90 mmHg, and/or the use of antihypertensive drugs. ^e^Dyslipidemia was defined as a low-density lipoprotein cholesterol level ≥140 mg/dL, high-density lipoprotein cholesterol level <40 mg/dL, triglyceride level ≥150 mg/dL, or the use of antilipidemic medications. BMI, body mass index; eGFR, estimated glomerular filtration rate; HbA1c, glycated hemoglobin; SBP, systolic blood pressure; DBP, diastolic blood pressure

Variables	All	Maximum lifetime BMI (kg/m^2^)			*P* for trend
		<26.7	26.7-32.0	≥32.0	
Number	63	21	21	21	
Crossing capillaries^a^ (%)	62.3 ± 11.4	60.8 ± 11.0	60.7 ± 11.8	65.3 ± 11.2	0.286
Age (years)	62.9 ± 8.9	65.9 ± 8.3	63.0 ± 6.2	59.9 ± 11.0	0.036
Female (%)	47.6	47.6	57.1	38.1	0.540
Smoking: former (%)	30.2	42.9	28.6	19.0	0.095
Regular exercise: absence (%)	42.9	33.3	52.4	42.9	0.536
Duration of diabetes (years)	13.6 ± 8.5	15.4 ± 9.3	14.0 ± 8.2	11.5 ± 7.8	0.198
Insulin (%)	23.8	33.3	14.3	23.8	0.472
Renin-angiotensin-aldosterone system inhibitor use (%)	49.2	38.1	52.4	57.1	0.221
Lipid-modifying medication use (%)	60.3	47.6	61.9	71.4	0.118
HbA1c (%)	7.4 ± 1.0	7.5 ± 1.0	7.2 ± 0.9	7.3 ± 1.1	0.536
eGFR (mL/min/1.73 m^2^)	68.2 ± 25.1	70.5 ± 26.2	67.6 ± 17.3	66.6 ± 31.0	0.757
Diabetic nephropathy^b^ (overt nephropathy or kidney failure) (%)	17.5	23.8	9.5	19.0	0.687
Diabetic retinopathy^c^ (%)	33.3	33.3	28.6	38.1	0.745
SBP (mmHg)	131.3 ± 15.6	131.3 ± 17.4	126.9 ± 13.7	135.7 ± 14.8	0.391
DBP (mmHg)	80.5 ± 9.8	76.4 ± 11.5	79.2 ± 8.3	85.8 ± 7.0	0.001
Hypertension^d^ (%)	68.3	57.1	66.7	81.0	0.100
Dyslipidemia^e^ (%)	77.8	71.4	76.2	85.7	0.269
BMI at the time of the survey (kg/m^2^)	26.5 ± 4.9	23.1 ± 2.5	25.5 ± 3.3	30.9 ± 5.0	<0.001
Waist circumference at the time of the survey (cm)	94.2 ± 12.7	87.3 ± 8.4	91.4 ± 10.2	103.9 ± 12.9	<0.001

The multiple linear regression analysis revealed that increased maximum lifetime BMI was significantly associated with the percentage of crossing capillaries, after adjusting for age, sex, regular exercise, duration of diabetes, HbA1c level, DBP, renin-angiotensin system inhibitor use, lipid-modifying medication use, and BMI or WC at the time of the survey (unstandardized regression coefficients: 0.47, *P* = 0.026; 0.37, *P* = 0.045; respectively) (Tables 2-3). Alternatively, BMI or WC at the time of the survey showed no significant association with the percentage of crossing capillaries (standardized regression coefficients: -0.21, *P* = 0.381; -0.04, *P* = 0.828, respectively) (Tables 2-3). The maximum lifetime BMI model diagnostics confirmed the assumptions of the multiple linear regression, including the absence of multicollinearity (variance inflation factor < 5), homoscedasticity, and normality of residuals, thereby ensuring the robustness of our results.

**Table 2:** Association of percentage of crossing capillaries with maximum lifetime BMI adjusted for age, sex, regular exercise, duration of diabetes, HbA1c level, DBP, renin-angiotensin system inhibitor use, lipid-modifying medication, and BMI at the time of the survey. BMI, body mass index; CI, confidence interval; HbA1c, glycated hemoglobin; DBP, diastolic blood pressure

Variables	Univariate analysis		Multivariate analysis		
	Unstandardized coefficients (95%CI)	*P*-value	Standardized coefficients	Unstandardized coefficients (95%CI)	*P*-value
Maximum lifetime BMI (per 1 unit)	0.54 (0.04-1.04)	0.036	0.47	0.96 (0.12-1.79)	0.026
Absence of regular exercise (vs. presence)	4.20 (-1.53 to 9.93)	0.148	0.23	5.22 (-1.05 to 11.48)	0.101
Duration of diabetes (per year)	-0.12 (-0.46 to 0.23)	0.498	-0.21	-0.28 (-0.68 to 0.11)	0.158
BMI at the time of the survey (per unit)	0.24 (-0.35 to 0.82)	0.421	-0.21	-0.48 (-1.56 to 0.61)	0.381
Lipid-modifying medication use (vs. none)	-4.65 (-10.43 to 1.13)	0.113	-0.16	-3.65 (-10.71 to 3.41)	0.304
DBP (per unit)	-0.02 (-0.31 to 0.28)	0.899	-0.14	-0.16 (-0.53 to 0.21)	0.385
Female (vs. male)	0.96 (-4.81 to 6.74)	0.739	0.04	0.97 (-5.46 to 7.40)	0.764
Renin-angiotensin system inhibitor use (vs. none)	-0.62 (-6.39 to 5.15)	0.831	0.04	0.95 (-5.89 to 7.79)	0.781
Age (per year)	0.06 (-0.26 to 0.39)	0.698	0.04	0.05 (-0.36 to 0.46)	0.821
HbA1c (per unit)	-0.50 (-3.37 to 2.37)	0.727	0.03	0.38 (-2.85 to 3.60)	0.816

**Table 3:** Association of percentage of crossing capillaries with maximum lifetime BMI adjusted for age, sex, regular exercise, duration of diabetes, HbA1c level, DBP, renin-angiotensin system inhibitor use, lipid-modifying medication, and waist circumference at the time of the survey. BMI, body mass index; CI, confidence interval; HbA1c, glycated hemoglobin; DBP, diastolic blood pressure

Variables	Univariate analysis		Multivariate analysis		
	Unstandardized coefficients (95%CI)	*P*-value	Standardized coefficients	Unstandardized coefficients (95%CI)	*P*-value
Maximum lifetime BMI (per unit)	0.54 (0.04-1.04)	0.036	0.37	0.75 (0.02-1.48)	0.045
Absence of regular exercise (vs. presence)	4.20 (-1.53 to 9.93)	0.148	0.23	5.63 (-0.64 to 11.90)	0.077
Duration of diabetes (per year)	-0.12 (-0.46 to 0.23)	0.498	-0.21	-0.27 (-0.67 to 0.13)	0.175
DBP (per unit)	-0.02 (-0.31 to 0.28)	0.899	-0.14	-0.19 (-0.56 to 0.17)	0.296
Lipid-modifying medication use (vs. none)	-4.65 (-10.43 to 1.13)	0.113	-0.13	-3.08 (-10.19 to 4.04)	0.390
Renin-angiotensin system inhibitor use (vs. none)	-0.62 (-6.39 to 5.15)	0.831	0.04	1.60 (-5.13 to 8.33)	0.635
Age (per year)	0.06 (-0.26 to 0.39)	0.698	0.07	0.09 (-0.32 to 0.50)	0.665
Female (vs. male)	0.96 (-4.81 to 6.74)	0.739	0.05	1.15 (-5.34 to 7.63)	0.724
Waist circumference at the time of the survey (per unit)	0.12 (-0.11 to 0.34)	0.314	-0.04	-0.04 (-0.38 to 0.31)	0.828
HbA1c (per unit)	-0.50 (-3.37 to 2.37)	0.727	0.02	0.20 (-3.03 to 3.43)	0.900

## Discussion

This study was conducted to investigate the relationship between maximum lifetime BMI and the percentage of crossing nailfold capillaries in patients with T2DM. Our findings showed that increased maximum lifetime BMI may increase the percentage of crossing nailfold capillaries, even after adjusting for potential confounding factors such as age, sex, regular exercise, duration of diabetes, HbA1c level, DBP, renin-angiotensin system inhibitor use, lipid-modifying medication, and obesity status at the time of the survey. A study on the relationship between current obesity status and the percentage of crossing nailfold capillaries in patients with T2DM revealed that current obesity status (abdominal obesity) is associated with an increased percentage of crossing capillaries [16]. However, our study showed that maximum lifetime BMI has a stronger influence on the increase in the percentage of crossing nailfold capillaries than current obesity status. The mechanism by which maximum lifetime BMI exerts this greater influence remains unclear. However, the crossing of nailfold capillaries is believed to occur owing to the loss of pericytes [16], which envelop blood vessels and provide stability to the vessel walls [27]. Moreover, a review of animal experiments on the relationship between obesity

and capillary rarefaction suggests that capillary rarefaction also results from the loss of pericytes, and its extent depends on the duration and severity of obesity [28]. Maximum lifetime BMI serves as a summary measure of obesity history [2], and typically, increased maximum lifetime BMI correlates with longer duration and greater severity of obesity [29]. Therefore, maximum lifetime BMI, reflecting the duration and severity of past obesity, might have had a stronger impact on the increase in the percentage of crossing capillaries, compared with obesity status at the time of the survey.

The present study suggests that past obesity may exhibit a long-term impact on microvascular health, regardless of current obesity status. In patients with obesity, microvascular dysfunction may improve following successful weight reduction surgery [30,31]. However, a prospective cohort study examining the effects of weight reduction surgery on microvascular function in patients with morbid obesity revealed that while weight loss significantly improved microvascular function in patients without metabolic syndrome, no improvement was observed in those with metabolic syndrome [32]. Our study participants were patients with T2DM, more than half of whom had metabolic syndrome. These findings suggest that in a physiological environment already characterized by factors such as hyperglycemia and hypertension, fully reversing the effects of past obesity may not be feasible, regardless of current obesity status. Given that the relationship between hyperglycemia, hypertension, and microvascular changes is interconnected and forms a vicious cycle [8], the observed association between maximum lifetime BMI and microvascular changes in this study provides insights into how past obesity may be linked to an increased risk of diabetic complications.

In three studies involving Japanese individuals with T2DM, both past and current obesity were associated with an increased risk of microvascular complications [4,5,33]. However, the present study did not show any association between maximum lifetime BMI and microvascular complications such as retinopathy or nephropathy. Potential explanations for this discrepancy could be the smaller number of patients included in this study compared with those in previous studies and variations in the extent and timing of the effects of obesity by organ [30,34].

Nailfold capillaries can be observed non-invasively, enabling patients to visualize these changes [17]. Diabetic complications often progress insidiously until reaching advanced stages [11]. Therefore, illustrating to patients the physiological changes resulting from past obesity, such as alterations in nailfold capillaries, may provide a tangible understanding of how obesity impacts their vascular health before the onset of diabetic complications. This approach may enhance patient adherence to diabetes management and lifestyle modifications, potentially mitigating the risk of future complications.

Our study has some limitations. First, given its cross-sectional nature, we could not delineate the temporal trajectory linking maximum lifetime BMI with crossing fingernailfold capillaries. Second, the duration of obesity should be considered a significant factor influencing outcomes; however, we only had data on maximum lifetime BMI, BMI, and WC at the survey, and not on the duration. Third, this study may be subject to selection bias, as individuals with obesity are potentially more likely to have their maximum lifetime BMI documented in their medical records. Fourth, the relatively small sample size of 65 participants, selected from an initial cohort of 126, may limit the precision of the estimates and the generalizability of the findings to broader populations. Nevertheless, accumulating evidence from multiple studies, even with imprecise estimates, is essential for developing a more comprehensive understanding of causal relationships [35]. Fifth, while this study focused on the macroscopic assessment of nailfold capillary abnormalities, the inclusion of molecular markers could provide deeper insights into the mechanisms linking maximum lifetime BMI, microvascular changes, and diabetic complications. Therefore, future studies should explore these molecular pathways to better elucidate the causal relationships. Finally, our research was confined to an Asian population, implying that the conclusions drawn may not universally apply to diverse ethnicities or geographical locales.

Despite these limitations, this study’s strengths include its focus on maximum lifetime BMI, emphasizing the long-term impact of past obesity on microvascular health. We adjusted for confounding factors that could potentially affect the relationship between maximum lifetime BMI and percentage of crossing nailfold capillaries, such as age, sex, obesity status at the time of the survey, and other diabetes-related factors. Additionally, the use of a standardized quantitative assessment of nailfold capillaries ensured highly reliable data.

## Conclusions

Maximum lifetime BMI was associated with an increased percentage of crossing capillaries in patients with T2DM, rather than obesity status at the time of the survey. These results emphasize the importance of lifelong weight management in the prevention of T2DM and its complications. They also highlight the necessity of considering maximum lifetime BMI alongside current BMI in the management of patients with T2DM. Larger prospective studies with more information are necessary to clarify the time-related aspects of this association.
